# COVID-19 Responses of South Korea as Hybrids of Governance Modes

**DOI:** 10.3389/fpubh.2021.654945

**Published:** 2021-09-17

**Authors:** Sora Lee, Ryan Wong

**Affiliations:** ^1^Menzies Centre for Health Governance, School of Regulation and Global Governance (RegNet), Australian National University (ANU) College of Asia & The Pacific, The Australian National University, Canberra, ACT, Australia; ^2^Department of Political Science, University of Munich, München, Germany

**Keywords:** COVID-19, hybrid governance, market governance, network governance, South Korea

## Abstract

The countries worldwide have adapted diverse governance approaches to the pandemic to suit their contexts. While the diversity of the country-specific governance responses has been widely discussed, the hybrids nature of those governance practices has been explored less. This study analyses the responses toward COVID-19 in South Korea as responsive dialogues of different modes of governance, i.e., consensus-based hierarchy, state-sponsored market, and principle-based network. This study aims to remind us that pandemic governance needs to enable organic and responsive processes for all actors in society. This conceptual discussion of the governance modes illustrates that the pandemic allowed the emergence of the hybrids of governance modes to cope better with the complex realities of the diverse sectors and actors in South Korea. The characteristic of the responses diverges from the conventional governance classification of or market-based. It is a responsive and evolving dialogue of different modes of governance. It would be productive to think beyond the oversimplified understandings of governance modes and embrace flexible and different hybrids of governance modes to be more responsive, effective, efficient, and equitable.

## Introduction

The pandemic responses of South Korea have been held up as the role model across the world. The defining feature is its proportionate and effective response that has not called for harsh lockdown measures similar to those which we have seen in decentralized federalist Western democracies such as Germany and Switzerland. What kind of institutions enables such an achievement? A survey of the pandemic policy demonstrates the value of strong public institutions (1), especially when long-term investment, trust toward the government (2), and fast adaptation of responses (3) are present. We agree with these assessments and distilled these observations into three governance principles that underpin the pandemic policies: resilience, efficiency, and transparency. More importantly, we move away from painting the government as the hero that took decisive actions or a villain that could exert authority over the culturally obedient citizens (4).

In this article, the story is justifiably complicated through the lens of the whole-of-society approach, whereby the interactions across the public, private, and civil society sectors should be the focus [(5), p. 4]. Their interactions have not been explicitly outlined in the nascent literature on the governance of COVID-19. This study draws the data from public source data and articles from March 1 to September 30, 2020, to discuss the early governance responses for COVID-19 in South Korea. We outline three governance enablers that may explain the successful containment of the disease in the early stage of the outbreak. The literature has acknowledged the shift from government to governance (6). It makes explicit governance modes, namely, hierarchy, market, and network (7). Hierarchy is replaced by the network (8). Hierarchy can also complement the network (9). Instead of the individual governance modes, most recent analyses focus on the interactions between the modes under the frameworks of hybrids governance (10) and meta-governance (11). The former has embraced principles of complexity and evolution while the latter was further developed into concrete typology around the relationship between the meta-governor and network governance (12). At the heart of these concepts is the understanding of balancing across governance modes. This article makes explicit how the modes complement each other in three systems that produce the responsive management of COVID-19 in South Korea to which the whole world has aspired.

## Consensus-Based Hierarchy

The modifications made to the Infectious Disease Control and Prevention Act (IDCPA) had a prominent influence in preparing South Korea for the pandemic scenarios. The IDCPA endows the government with specialized structures for distributing resources and mobilizing various actors across the whole society in the effort to fight against the spread of infectious disease (13). IDCPA was enacted to stipulate certain powers and responsibilities of the state, local governments, private sectors, medical personnel, and the public. It permits a wide range of regulations including the basic plans and projects for prevention and surveillance governance of infectious diseases, intergovernmental protocols in crisis situations, public-private response, process of the announcement and reporting on diseases, epidemiological tracing investigations, preventive measures, and compensations (14).

In the modified version, IDCPA legitimizes the central roles and functions of the Korean Centers for Disease Control and Prevention (KCDC) during the pandemic. The centralization of power in the hands of authority for responding to crises is not uncommon. After Middle East Respiratory Syndrome (MERS), the KCDC acquired greater capacity through increased staffing and training, particularly in epidemiology. Specialized divisions have been established for risk assessment, emergency operations, crisis communication, and partner coordination (15, 16). The KCDC is authorized to coordinate with the newly established subnational centers for epidemic countermeasures across provincial and municipal governments and specialized hospitals.

Silos in bureaucratic administration are likely to make coordination of crisis response inefficient when time and timing are crucial. The gravity of the crisis has triggered several administrative reforms to the management and approval systems. According to KCDC (17), the smart management system (SMS) enables mass tracing of individuals who have a positive diagnosis or have interactions with infected individuals. It is known as the COVID-19 SMS. The government conducts epidemiological tracing on a single data platform to reduce administrative inefficiencies across multiple jurisdictions. KCDC runs the contact tracing system which uses data from 28 organizations such as the National Police Agency, the Credit Finance Association, three smartphone companies, and 22 credit card companies to trace the movement of infected individuals with a processing speed of 10 min. This speedy tracing allows the KCDC to inform the local public health center, which will then notify the infected individuals.

The coordination through this SMS has been made possible due to high level of digitalization in South Korea, which has the highest number of cashless transactions in the world, as well as transportation cards that records all the destinations and are compatible with all transportation means. South Koreans also have the highest phone ownership rates in 2019 (18), and the phone companies require customers to register with their authentic identification by law. As a safety measure, only epidemic investigators at KCDC can access the location information, and once the COVID-19 outbreak is over, the personal information used for the tracing of contacts will be removed.

Neither Singapore, South Korea nor Hong Kong reveals names of the infected individuals but the combination of the information being disclosed, together with other information in the public domain, may potentially allow the speculation of identification. There have been few accounts of illegal doxing in early February, where identities of the infected individuals and personal data (gathered from public and private sources) have been disclosed on websites, social media, and public forums without their consent (19). On March 15, the government has announced a new guideline for privacy protection and banned the release of any specific information that might be used to identify infected individuals, but varying degree of information release was practiced among different local governments (20). At the Ministry of Land, Infrastructure, and Transportation and KCDC online briefing of SMS, public managers present at the briefing shared their cautions and promised vigilant monitoring of the system (21).

## State-Sponsored Market

Experts in the field recognize that after the MERS outbreak in 2015, there have been various changes in the infectious disease governance of South Korea. The main change is realizing that the state of medical care and quarantine are two separate affairs. The medical facility with state-of-the-art medical knowledge and technologies has failed to quarantine citizens when MERS became extremely contagious. As Lee (17) points out, there has been a significant portion of the budget spent on R&D, amounting to ~49% of the total infectious disease governance budget. After the MERS crisis, experts realized that it was imperative to have test kits as early as possible because the development of treatments or vaccines is expected to take a significant time. Therefore, the government took an initiative in R&D with the biotech industry to develop the necessary technology for early and mass diagnosis.

In 2016, the budget on contagious diseases and quarantine systems has been expanded 134% compared to the previous year, a jump from ~US$59–137 million. In 2020, it has continuously risen to US$166 million, which is a 182% rise for the last 5 years (17). During 2019 and 2020, crisis management and collaborative governance infrastructure for different national, local, and international authorities have been established, enabling a nationwide epidemiological tracing platform that South Korea is using now.

The single most highlighted government response from the media was the emergency fast track approval. The public sector took a decisive measure to stop the virus using emergency fast track approval of the test kits. Experts were called to meetings with government officials and acted as a boundary spanner between the private and public sectors, communicating the support and the sense of urgency by KCDC to biotech companies that specialize in test kit development. The knowledge on the virus that KCDC has so far has been shared. A week after the meeting, KCDC approved the diagnostic test of one company. KCDC decided to rapidly inspect tests by releasing them to labs, then cross-check to evaluate their accuracy. Many more prototype tests followed, and health officials were well-armed to attack a fast-moving virus with aggressive mass testing. More than 2,301,303 people have been tested (as of September 28) (22). This, in turn, has allowed the biotech industry to share abundant samples to improve the accuracy of test kits. Korea can conduct up to 15,000–20,000 tests a day, and there was enough production to export test kits to other countries.

The government has cultivated R&D-based bioventures with strong political will and vision for the global market. They are used to fund R&D projects specifically on vaccines, preventive technologies, and test kits. Among those technologies, the government of Korea has emphasized the development of polymerase chain reaction test kits for fast, accurate, and mass testing. The total R&D projects amount to US$68 million, allocated for the prevention and diagnosis of contagious diseases in 2020 (17).

## Principle-Based Network

The national briefings and policy documents emphasized its vibrant communication with the public. The legal basis for sharing the latest available scientific information was stipulated in IDCPA, which establishes the right of the public to be informed about the latest developments and responses to outbreaks and infection control. Experts in the field have participated in sharing accurate information by actively addressing “fake news” in a variety of media platforms. Citizens also have gathered online to generate accurate information on available masks.

Central Incidence Management System for Novel Coronavirus Infection (IMS) discussed ways to eradicate fake news that groundlessly aggravates the fear and halt of public its creation at its source by sharing accurate information and ensuring fact-checking (23). Relevant government bodies and ministries such as the Korea Communications Commission; Ministry of Health and Welfare; Ministry of Culture, Sports and Tourism; and National Police Agency have decided to establish a new response system to identify fake news lacking factual grounds and promptly inform telecommunication services and Internet service providers. The Korea Communications Commission will also call emergency meetings for deliberating on fake news cases. IMS highlighted the need for all press organizations to ensure accuracy in their reporting and stressed its determination to block the spread of fake news by cooperating with telecommunication services and Internet service providers and sharing the reliable information of the government promptly. Police in South Korea are investigating a rise in false rumors about the coronavirus, including a scam in which people are asked to provide personal details in return for access to information about the spread of the disease. There will be a cyber unit of the national police agency to exclusively deal with “fake news,” which leads to excessive public anxiety and causes confusion in infection control.

Media appearance of experts is frequent, in alignment with the strategy of Korea to strictly respond to fake news, which may contribute toward unnecessary anxiety and confusion in public regarding the control of COVID-19. For instance, experts from the Korean Federation of Science and Technology Societies, the National Academy of Medicine of Korea, and the National Research Council of Science & Technology appear actively on online media platforms to diffuse information and held an online forum to fact check information related to COVID-19 (24). Doctors from private hospitals who have been fighting the disease at the front line actively appear on TV and other media platforms to address the confusion of the public and misunderstandings of the disease.

After the emergence of the *Itaewon* cluster in May, several media emphasized the nature of the clubs and the super spreader, triggering homophobic responses from the public. This reflects the still conservative nature of the country and prejudices which restrict sexual minorities to be integrated into society (25). Various organizations acting for the rights of lesbian, gay, bisexual, and transgender (LGBT)+ groups have formed a queer action against COVID-19 center to work together with KCDC and Seoul Metropolitan city government to encourage those who are still not tested, criticize discriminatory media coverages, and campaign against potential exposure to domestic violence and discrimination in the workplace after being tested (26).

As we can see from the responses toward the LGBT community, the whole-of-society efforts, there is an inherent disparity of multiple actors in the society of Korea that are amplified during the crisis of COVID-19. There have been physical attacks and online harassment toward the group of infected individuals, namely, Chinese immigrants, Christians with unorthodox faiths, and LGBT people have occurred. This is in part due to the level of disclosure of personal information, which is age, gender, and workplace. This has been carefully decided by the government for the greater public good, nonetheless, some have been using these details to narrow down those who are infected on social media, putting them at risk of discrimination. Furthermore, the authority has taken punitive measures for those who do not come forward for testing despite the possibilities of infection. However, it would mean greater risk for those who are already in discriminative social contexts. Thus, the rights of vulnerable groups need to be mindfully considered to achieve whole-of-society measures. Students and their families are affected by school closure decisions during stage 2 of the social distancing period. KCDC and local authorities have put priority tracing for potential infected cases in the school environment to make the school closures as brief as possible. While the control for the spread is imminent, authorities have expressed the understanding that prolonged school closure would place an extreme burden on students and parents in their daily lives. Emergency care support for vulnerable families, a systematic online curriculum for potential prolonged school closure, and the inclusion of the circumstances of individual schools in the decision-making process for school closures have been suggested by Congress and the government, and are being implemented (27). Public health and the freedom to lead a life as one values need to be weaved intricately with the agile, competent, and transparent government and responsible citizens (28).

## Discussion

The main idea in this article is to help readers and thinkers break free from the rigid framework of governance modes. The pandemic response of South Korea demonstrated that the modes overlap, and the analysis of governance as hybrids are closer to reality. These hybrids include: (1) the state that undertakes coordination based on the consensus of actor-networks, (2) the market that is repurposed with a high-risk investment of the state, and (3) the network that is steered by traditional principles of public governance. Informed by such flexible and nuanced analysis, the future debates on governance should move beyond the oversimplified division of interventionist state vs. market deregulation. An effective, efficient, and equitable pandemic response will call for the best features of the hierarchy, market, and network in different hybrid forms for achieving different purposes at different times.

## Data Availability Statement

The original contributions presented in the study are included in the article/supplementary material, further inquiries can be directed to the corresponding author/s.

## Author Contributions

SL and RW have contributed equally to the conceptualization of the article and revised the article together. SL has drafted the article. RW added his insights. Both authors contributed to the article and approved the submitted version.

## Funding

SL was supported by the Australian Postgraduate Award (APA) for her current doctoral program at ANU.

## Conflict of Interest

The authors declare that the research was conducted in the absence of any commercial or financial relationships that could be construed as a potential conflict of interest.

## Publisher's Note

All claims expressed in this article are solely those of the authors and do not necessarily represent those of their affiliated organizations, or those of the publisher, the editors and the reviewers. Any product that may be evaluated in this article, or claim that may be made by its manufacturer, is not guaranteed or endorsed by the publisher.
